# Portable Molecularly Imprinted Polymer Sensor for Rapid Swab‐Based Detection of Norovirus

**DOI:** 10.1002/adhm.71467

**Published:** 2026-07-21

**Authors:** Amy Dann, Pankaj Singla, Minji Kim, Sloane Stoufer, Clare Thomas, Robert D. Crapnell, Craig E. Banks, Jake McClements, Shayan Seyedin, Mark Geoghegan, Samuel T. Jones, Christopher F. Blanford, Matthew D. Moore, Marloes Peeters

**Affiliations:** ^1^ Department of Chemical Engineering, Nancy Rothwell Building University of Manchester Manchester UK; ^2^ School of Engineering, Merz Court Newcastle University Newcastle Upon Tyne UK; ^3^ Department of Food Science University of Massachusetts Amherst Massachusetts USA; ^4^ School of Chemistry University of Birmingham Edgbaston Birmingham UK; ^5^ Faculty of Science and Engineering, Dalton Building Manchester Metropolitan University Manchester Great Britain; ^6^ Manchester Institute of Biotechnology The University of Manchester Manchester UK

**Keywords:** biomimetic sensor, electrochemical detection, food safety, molecularly imprinted polymer, norovirus, public health

## Abstract

Norovirus poses a continuous challenge for healthcare systems, leading to increased hospital admissions, infection‐control demands, and costly outbreak management. Gold‐standard diagnostics such as real‐time reverse transcriptase polymerase chain reaction (RT‐PCR) require specialized laboratory equipment and trained personnel. This work reports a portable electrochemical sensor based on an electropolymerized molecularly imprinted polymer fabricated directly on an electrode surface for rapid norovirus detection using voltametric read‐out. The sensor was tested against four norovirus virus‐like particle (VLP) genotypes that each exhibited consistent responses across a broad concentration range. Sensor specificity and selectivity were demonstrated through comparison with a nontarget imprinted control polymer and testing against four nontarget live viruses. The imprinted polymer electrodes showed excellent stability, with no significant loss of performance after storage under ambient conditions for up to three months. For practical applicability, nitrile gloves were inoculated with each VLP genotype and sampled using standard swabbing procedures. The entire process was completed in ∼ 15 min, with electrochemical acquisition completed in 20 s. Successful detection of each genotype was achieved with detection limits between 154 and 279 fg/mL (8.78 × 10^3^ and 1.59 × 10^4^ particles/mL) for swab samples. This rapid and portable platform supports near real‐time norovirus screening at the point‐of‐care.

## Graphical abstract

This work reports a rapid, portable smartphone‐based sensor capable of detecting four norovirus genotypes from swab samples within 15 min. Sensor selectivity was validated through evaluation against four common live viruses. The swab‐based sampling process enabled simple sample preparation combined with the compact detection system, supporting use of the sensor outside laboratory settings.

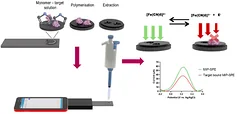

## 1 Introduction

Norovirus is the leading cause of acute gastroenteritis, accounting for 18% of cases [1]. The consequences extend beyond individual illness as it can place additional strain on healthcare systems. As one of the most widespread foodborne pathogens, norovirus is responsible for 677 million infections and results in more than 200,000 deaths globally each year [2]. Individuals are estimated to experience three to eight norovirus infections over their lifetime, with at least one occurring before the age of five. Although typically self‐limiting, norovirus infections have significant societal and healthcare implications [3]. Worldwide, norovirus infections result in US$4.2 billion in direct healthcare costs and US$60.3 billion in societal losses each year [4]. Within healthcare settings, norovirus poses a major infection control challenge, contributing to staff sickness, costly outbreak management, and unit closures. A survey of US hospitals reported that norovirus accounted for 65% of unit closures, a consequence that limits bed capacity, delays patient care, and places additional pressure on hospital resources [5, 6].

Real‐time reverse transcriptase polymerase chain reaction (RT‐PCR) remains the current gold standard for norovirus detection, as it enables the selective amplification of viral RNA to levels that can be reliably detected [7]. However, despite its sensitivity and specificity, RT‐PCR is not well suited for point‐of‐care testing due to the requirement for specialized equipment, trained personnel, and controlled reaction conditions [8]. Another challenge in norovirus detection arises due to the high genetic diversity of norovirus, which necessitates the use of multiple primers and probes to encompass the wide range of human‐infecting strains [9]. This diversity is exemplified by the genotype GII.4 that has been the dominant strain for many years; however, at the start of 2025, GII.17 was named the “Ferrari of viruses” due to a surge in cases and has since overtaken GII.4 as the most prevalent genotype in the US and other countries [10, 11, 12]. These epidemiological changes make it clear that a versatile sensor capable of capturing a broad spectrum of genotypes is essential for maintaining effective norovirus monitoring. Alternative diagnostics to PCR have been explored, including enzyme‐linked immunosorbent assays and a growing range of biosensors reported in literature [13, 14, 15, 16]. However, many of these approaches remain limited by their reliance on biological recognition elements or complex steps. These limitations have prompted interest in synthetic recognition elements that offer improved stability compared to their biological counterparts.

Molecularly imprinted polymers (MIPs) are synthetic materials containing recognition sites tailored to a specific target, mirroring biological molecular recognition. They are produced by polymerizing functional monomers around a template molecule that shapes the polymer network. Subsequent removal of the template leaves complementary cavities behind that can then selectively bind to the template molecule [17]. MIPs are particularly well suited for norovirus detection as their adaptable design can accommodate the extensive genetic and structural diversity of norovirus genotypes, and can be readily reconfigured as new genotypes emerge. This flexibility arises from the wide choice of synthesis methods, functional monomers, and template choices available, enabling the development of polymers tailored to specific application needs [18, 19]. For the development of electrochemical sensors, electropolymerization offers a convenient synthesis approach by synthesizing the polymer directly onto the working electrode surface, bypassing the immobilization of the recognition element [20, 21]. Pyrrole was selected because it is the most widely used monomer in electropolymerization‐based imprinting due to its ease of electropolymerization under mild conditions and its long‐term stability, making it particularly suitable for imprinting of macromolecules [22]. Moreover, the charged sites on pyrrole support hydrogen‐bonding and electrostatic interactions with norovirus residues, thereby facilitating specific binding.

Integrating MIPs into portable biosensors presents a promising strategy to mitigate pathogen transmission, particularly as norovirus contamination can occur at any stage of the ‘farm‐to‐table’ process, thus allowing monitoring throughout [23]. In recent years, a range of MIP‐based portable biosensors have been developed for food safety to complement the established laboratory‐based methods [24, 25, 26]. These platforms offer many advantages such as reduced testing times, minimal sample preparation, and the ability to integrate with user‐friendly platforms, bringing testing outside specialized laboratories. Alongside this, MIP‐based sensors for viral pathogen detection have been explored, including SARS‐CoV‐2 [27, 28], tobacco mosaic virus [29], and norovirus [30, 31]. A notable example of multiplexed virus detection was reported by Luo et al., who developed a multifunctional fluorescence‐based MIP sensor capable of detecting hepatitis A and hepatitis B. In this approach, separate virus‐specific imprinted polymers were prepared for each target virus and combined within a single sensing platform, enabling dual‐virus detection through distinct fluorescence responses [32]. Although this work demonstrated the potential for multiplexed MIP‐based sensing for virus detection, it still required two separate MIPs contributing to further synthesis steps.

Here, we present a novel strategy for portable norovirus detection, employing an electropolymerized pyrrole MIP integrated with a compact electrochemical platform for rapid point‐of‐care norovirus detection. The norovirus protruding (P) domain was selected as part of this capsid protein that forms the outer surface of the virus. In this work, the sensor is evaluated against multiple targets, including four norovirus virus‐like particles (VLPs) and several live nontarget viruses, demonstrating its selectivity toward norovirus genotypes. For practical applicability, the focus is placed on swab samples collected from gloves. In food‐handling and clinical environments, gloves constitute a high‐risk contact interface, frequently interacting with diverse surfaces and acting as potential vectors for cross‐contamination, thereby providing an informative target for environmental monitoring. While our previous work has explored the use of nanoMIPs combined with thermal and electrochemical methods, this work offers a simplified electropolymerisation‐based synthesis, swab‐based sample collection, and a twofold reduction in detection time from 30 to 15 min [30, 31]. This study presents a rapid platform for point‐of‐care norovirus detection, offering healthcare settings and the food industry a practical tool to enable proactive interventions, prevent transmission, and reduce the overall burden of the virus.

## 2 Results and Discussion

### 2.1 Experimental Conditions

**FIGURE 1:**
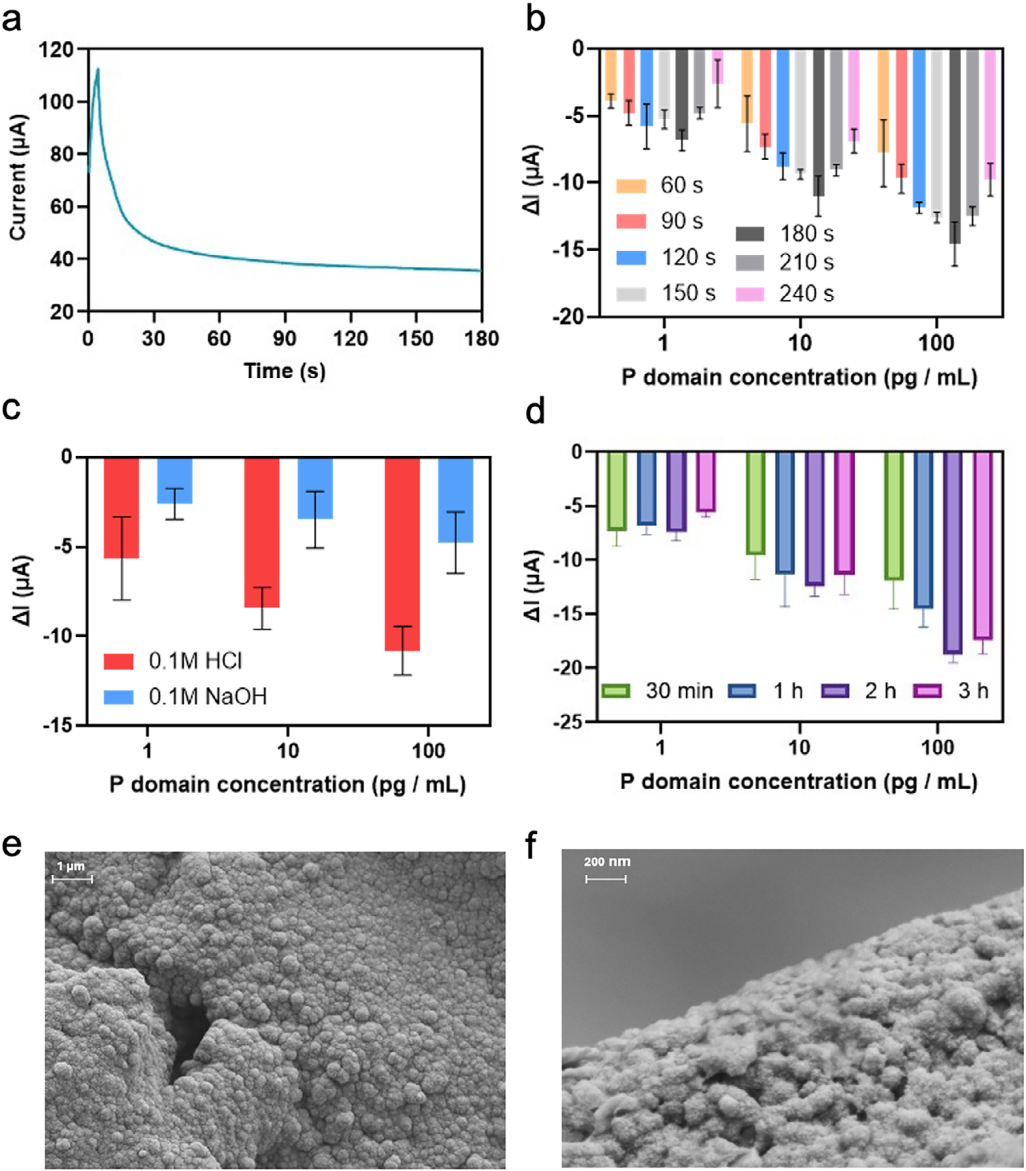
Optimization results. (a) Polymerization of pyrrole using chronoamperometry. (b) Influence of polymerization time on the response upon rebinding of the P domain. (c) Influence of extract buffer on rebinding. (d) Influence of extraction time on rebinding. The response is presented as mean ± SD (µA per log10[concentration]). Bars represent the mean with error bars showing the SD, n = 3. (e) SEM image of polypyrrole on the SPGE surface. (f) SEM side view image of polypyrrole on the SPGE surface.

To fabricate the MIP sensor, pyrrole was electropolymerized onto screen‐printed graphite electrodes (SPGEs) in the presence of the norovirus GII.4 P domain. The optimization of the MIP sensor was systematically evaluated through a series of electrochemical experiments. Pyrrole was selected because it combines intrinsic conductivity with aqueous solubility and low‐potential electropolymerization, enabling formation of a stable imprinted layer under conditions compatible with preservation of the P domain template [33]. A representative chronoamperometric curve recorded during the electropolymerization of pyrrole demonstrated a rapid initial current followed by an exponential decay, characteristic of polymer film growth on the electrode surface (Figure 1a). Optimal parameters were determined based on the greatest change in current (Δ*I*) upon rebinding of three concentrations of the P domain template (1, 10, 100 pg/mL) using cyclic voltammetry (CV). The impact of polymerization time on sensor performance was evaluated (Figure 1b), with the largest Δ*I* observed after 180 s, suggesting this duration yields optimal film thickness and binding site accessibility. The sensor response after template extraction in two conditions (0.1 M HCl vs. 0.1 M NaOH) demonstrated superior performance in acidic conditions (Figure 1c). Finally, Figure 1d demonstrates the effect of incubation time in the extraction buffer, showing no significant difference in sensor response between 30 min and 3 h, allowing for extraction to be performed quickly. The scanning electron microscopy (SEM) images (Figure 1e,f) show a uniform layer of polypyrrole covering the full working electrode. They also reveal a porous morphology, which facilitates the access of the redox couple (Fe (CN)_6_) to the electrode surface for current generation while simultaneously offering high surface area for the interaction of target molecules. X‐ray photoelectron spectroscopy (XPS) analysis (Figure S1 and Table S1) of the polypyrrole film showed a dominant sp^3^‐hybridized carbon component, along with a smaller sp^2^ contributions suggesting a partially conjugated polymer backbone. The N1 spectrum (Figure S1) confirms nitrogen incorporation and doping, with clear contributions from ‐NH and C─N+ indicating charged sites.

### 2.2 Template Rebinding and Control Polymer

**FIGURE 2:**
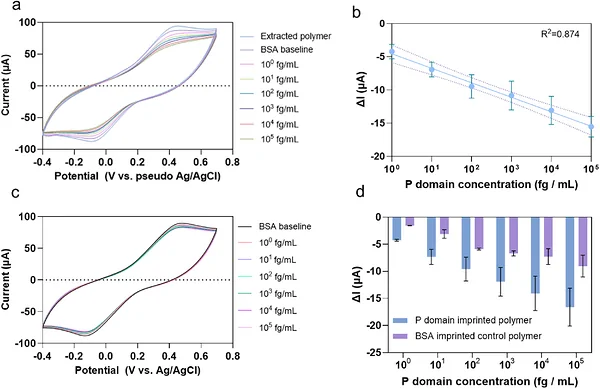
(a) Typical cyclic voltammogram showing the response of the imprinted polymer electrode following extraction, after BSA blocking and to increasing P domain concentrations. (b) Dose‐response of the sensor to increasing P domain concentrations. The response is presented as mean ± SD (µA per log10[concentration]), n = 3. Circular symbols represent the mean of three independent replicates, with error bars showing the SD of the measurements. Colored lines indicate the linear fit to the data, and dotted lines represent the 95% confidence interval. (c) Typical cyclic voltammogram of the BSA imprinted electrode after BSA blocking and exposure to P domain concentrations. (d) Comparison of the response of the target‐imprinted and control polymer to the P domain. Data are presented as mean ± SD, n = 3. P‐values were calculated using a paired t‐test, *p < 0.05, **p < 0.01.

Prior to analysis, the imprinted pyrrole electrode was incubated in a 1% bovine serum albumin (BSA) solution to minimize nonspecific binding. Subsequent rebinding studies were conducted by adding increasing concentrations of the P domain protein. A progressive decrease in the oxidation peak current response was observed, indicating successful and concentration‐dependent binding of the protein to the polymer (Figure 2a,b). This reduction in current can be attributed to the binding of the P domain to the imprinted sites, which hinders the diffusion of the redox couple to the electrode surface and thereby restricts electron transfer processes [34, 35]. To ascertain the effect of the imprinting process, a control polymer was produced by using BSA as a substitute for the P domain template during polymerization, while all other parameters remained unchanged. BSA (66 kDa) was selected due to its similar molecular weight to the P domain dimer (66 kDa), allowing for an evaluation of whether the sensor could differentiate between a nontarget protein of comparable size and the target analyte. A statistical comparison between the P domain imprinted polymer and the BSA imprinted polymer revealed a significant difference in response (p = 0.0012, t‐test), demonstrating that the imprinting process results in selective binding to the target (Figure 2c,d).

### 2.3 VLP Detection

**FIGURE 3:**
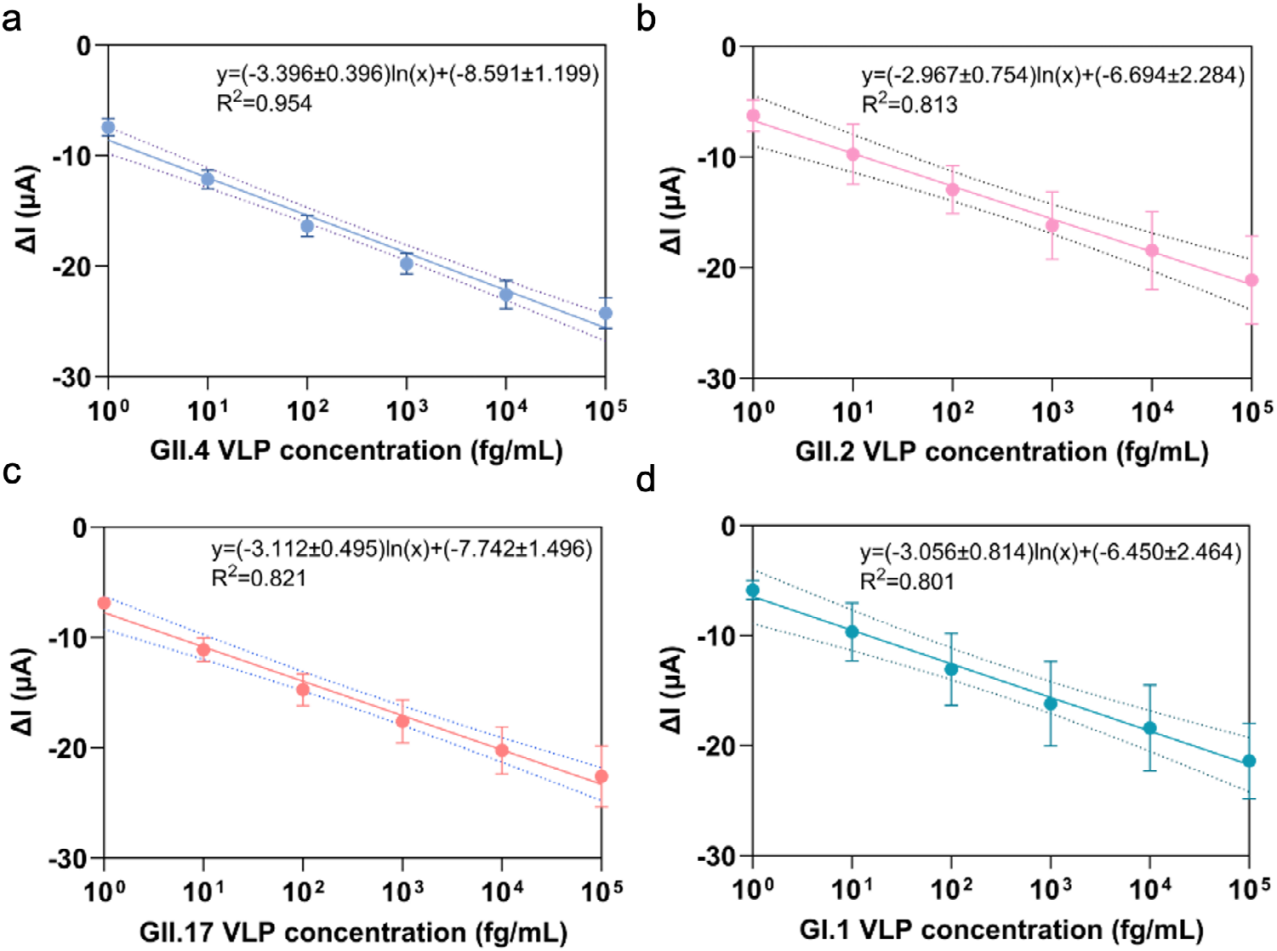
Dose‐response curves showing the Δ*I* upon the addition of VLP concentrations using CV. (a) GII.4 VLP. (b) GII.2 VLP. (c) GII.17 VLP. d) GI.1 VLP. The response is presented as mean ± standard deviation (SD) (µA per log_10_[concentration]), n = 3. Circular symbols represent the mean of three independent replicates, with error bars showing the SD of the measurements. Colored lines indicate the linear fit to the data, and dotted lines represent the 95% confidence interval.

The next stage in the study was to assess the ability to detect VLPs. These VLPs serve as noninfectious alternatives to the native virus and are composed of 90 dimers of the major capsid protein, which assemble into the icosahedral capsid structure with the P domain being the outermost portion of the capsid that interacts with ligands/receptors. Four norovirus genotypes—GII.4, GII.2, GII.17, and GI.1 – were tested across a concentration range of 1 to 10^5^ fg/mL (5.7 × 10^1^ to 5.7 × 10^6^ particles/mL). Each genotype produced a concentration‐dependent response across a broad concentration range (Figure 3). As expected, the GII.4 genotype exhibited the greatest signal response, reflecting the fact that the imprinted GII.4 P domain template used was derived from this genotype. Despite the preferential response toward GII.4, notable signal responses were also observed for GII.2, GII.17, and GI.1, suggesting that the binding cavities possess sufficient binding sites to accommodate other genotypes. This binding is likely to arise from conserved motifs within the more conserved P1 subdomain of the target P domain, which promote partial recognition across genotypes. It is interesting that, despite the hypervariable P2 subdomain also being targeted within the P domain, such broad reactivity was observed, suggesting that MIP‐based sensing may be especially advantageous for viral targets that exhibit high diversity. The limit of detection (LoD) for the four genotypes ranged from 105 – 209 fg/mL, corresponding to 5.99 × 10^3^ and 1.19 × 10^4^ particles/mL (Table S2). This cross‐reactivity is advantageous for practical diagnostic applications, as it enables the simultaneous detection of multiple genotypes in a single assay format, a traditional challenge for viruses as diverse as norovirus.

### 2.4 Shelf Life

A major limitation of many point‐of‐care devices is their use of biological receptors, which depend upon cold chain storage and often have a restricted shelf life, which collectively increase both the cost and logistical complexity of large‐scale deployment [36]. To evaluate the stability and storage robustness of the developed sensor, the imprinted electrodes were stored in a dark cupboard within a sealed container under ambient conditions for a period of up to three months. Each storage condition was assessed using a different stored electrode, ensuring that the reported responses were not affected by repeated use of the same sensor. Their performance was assessed at defined intervals of one week, one month, and three months by measuring the rebinding response of the P domain (Figure S2). Across the concentrations, no significant difference between the sensor response and the time in storage was observed. The consistent signal indicates that the polymer recognition sites retained their structural integrity and recognition capability, with minimal degradation or loss of binding functionality. The stability is likely to arise from the chemically crosslinked and inert nature of the polypyrrole that preserves the functionality of the cavities. This degree of stability under ambient conditions demonstrates a key advantage over biological recognition elements such as enzymes and antibodies that require strict storage conditions [37, 38]. This offers a major advantage for biosensor transport, as eliminating the need for cold‐chain storage reduces complexity and cost, enhancing accessibility and commercial viability [39].

### 2.5 Selectivity

To assess the selectivity of the norovirus‐targeted sensor, four viruses were analyzed, including herpes simplex virus type 2 (HSV‐2), cytomegalovirus (CMV), human coronavirus 229E (HCoV‐299E) and respiratory syncytial virus (RSV). The characteristics of these viruses are noted in Table S3 [40, 41, 42, 43, 44]. These viruses were chosen as they represent common human pathogens that can be present in the environment, posing potential sources of contamination in real‐world scenarios [45, 46, 47, 48]. It should be noted that the concentrations of the nontarget viruses were reported in PFU, which quantify infectious viral particles rather than the total number of viral particles present. For comparison purposes, a 1:1 PFU‐to‐viral particle ratio was assumed, representing a conservative estimate of the total particle concentration. In reality, viral preparations commonly contain a substantial proportion of noninfectious particles, meaning that the total number of particles exposed to the sensor is likely to be considerably higher than the reported PFU values. Therefore, the relatively low responses observed for the nontarget viruses may reflect sensor exposure to a much larger number of particles than indicated by the PFU concentration, further supporting the selectivity of the MIP toward norovirus. Paired t‐tests were used to compare the responses of GII.4 and each of the nontarget viruses at 10^3^, 10^4^, and 10^5^ particles/mL. The calculated values presented in Table S4 reveal statistically significant differences between the responses of the norovirus VLPs and each of the other tested viruses. At all measured concentrations, the magnitude of the Δ*I* of the VLPs was significantly greater than that of HSV‐2, CMV, HCoV‐299E, and RSV, with p‐values consistently below the significance threshold. This strong contrast in sensor response suggests minimal nonspecific binding, indicating the cavities formed during the imprinting process favor norovirus particles. The absence of cross‐reactivity toward these live infectious viruses is particularly encouraging, as real‐world samples may contain a diverse mixture of viral contaminants. These findings support the robustness and selectivity of the norovirus sensor under conditions relevant to practical monitoring. This work is limited by the reliance on PFU‐based measurements, which may underestimate total viral particle exposure; future investigations could improve upon this by directly quantifying total particle concentrations.

### 2.6 Square Wave Voltammetry

**FIGURE 4:**
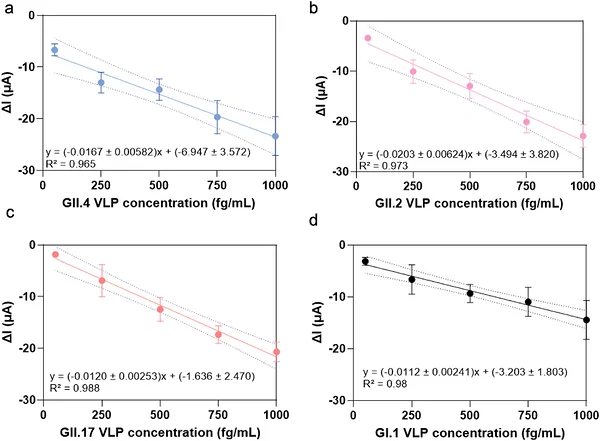
Dose‐response curves showing the ΔI upon the addition of VLP concentrations using SWV. (a) GII.4 VLP. (b) GII.2 VLP. (c) GII.17 VLP. (d) GI.1 VLP. The response is presented as mean ± SD (µA per log_10_[concentration]), n = 3. Circular symbols represent the mean of three independent replicates, with error bars showing the SD of the measurements. Colored lines indicate the linear fit to the data, and dotted lines represent the 95% confidence interval.

Focusing on maximizing the practicality and portability of the sensor system, the analysis method was transitioned from CV to square wave voltammetry (SWV), as this technique not only provides a much faster detection time (∼ 3 min to ∼20 s) but also simplifies data acquisition and interpretation by requiring only a single scan. The initial analysis confirmed the sensor's capability to detect the P domain with a concentration range of 50 to 1000 fg/mL, demonstrating the analysis method continued to allow detection at trace level, which is essential due to the low infectious dose of the virus (Figure S3). This approach was subsequently applied to all four VLPs, each of which elicited a measurable concentration‐dependent response (Figure 4). Similar to the CV results, LoD for the four genotypes ranged from 153–173 fg/mL, corresponding to 8.72 × 10^3^ and 9.86 × 10^3^ particles/mL (Table S2), confirming that SWV achieved comparable analytical performance to CV despite taking a fraction of the time. This underscores the suitability of SWV for rapid yet sensitive portable detection.

### 2.7 Detection of Norovirus VLPs From Surface Swabs

**FIGURE 5:**
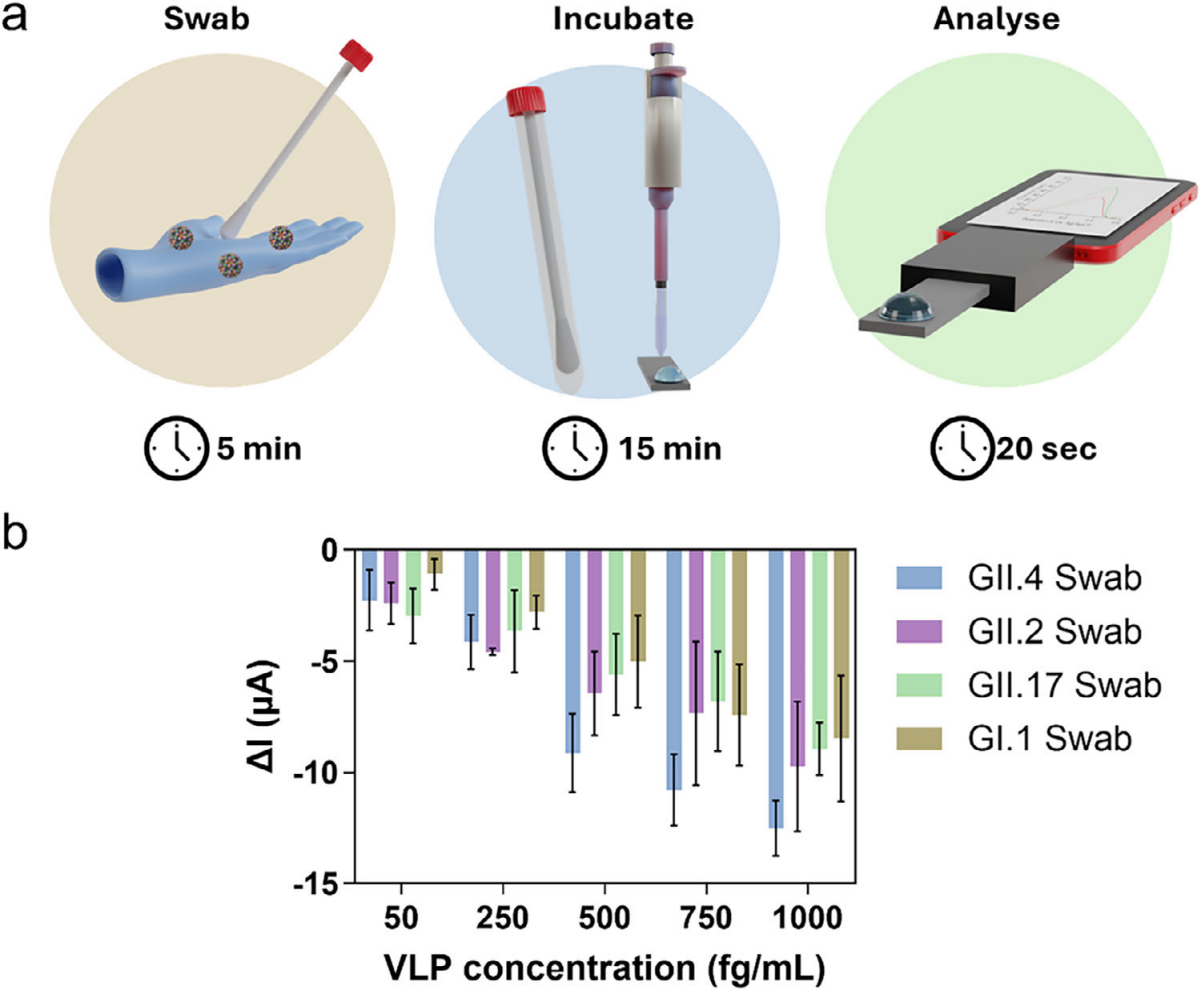
(a) Illustration of the three‐step workflow—swab, incubate and analyze. (b) The response is presented as mean ± SD (µA per log10[concentration]). Bars represent the mean with error bars showing the SD, n = 3.

To evaluate the real‐world applicability of the developed MIP‐based sensor, norovirus VLPs were detected following recovery from contaminated glove swabs (Figure 5). The workflow involved three main stages with the first step being the inoculation of VLPs and swabbing of a defined area of a nitrile glove. Next, the swab is added to phosphate buffered saline (PBS) (5 min) and a sample is incubated on the electrode surface (10 min). Finally, the electrochemical measurement can be performed using a smartphone with a compact potentiostat. The streamlined workflow was designed to simulate testing of norovirus‐contaminated gloves, whilst minimizing sample handling and instrument requirements, making it compatible with point‐of‐care detection. As shown in Figure 5, the sensor exhibited a clear suppression of the oxidation peak when exposed to the swab samples, indicating successful recovery of the VLPs from the glove surface. In line with the previous results, the LoD for the four genotypes ranged from 154–279 fg/mL, corresponding to 8.78 × 10^3^ and 1.59 × 10^4^ particles/mL (Table S2). The observed variability in responses, reflected in the larger error bars, can be attributed to differences in viral recovery from the glove surface. However, the primary objective of this sensor is not precise quantification but rather rapid identification of contaminated samples. Upon comparing the responses of each of the genotypes, t‐tests showed no statistically significant differences among the GII genotypes; however, a significant difference was observed between the GII genogroup and the GI.1 genotype. The observed difference between the GII genogroup and GI.1 likely reflects inter‐genogroup structural differences that become more evident in swab samples, which could be due to matrix effects and variable surface recovery.

Detecting contamination on a glove can serve as an indicator of potential cross‐contamination, supporting timely intervention and improved hygiene management. For a public health tool, versatility in sample collection and preparation is crucial. However, electrochemical detection of norovirus on food‐contact surfaces might be affected by various parameters ranging from the surface material to matrix effects due to interferents present on the surface (e.g., food/cleaning residues, proteins, fats, and bio‐film formation) to swab recovery efficiency. Future validation should therefore test representative food‐contact surfaces spiked with norovirus (or VLPs where appropriate) and benchmark the results of this sensor against the current state‐of‐the‐art (e.g., RT‐qPCR). While this study focuses on swabbing a nitrile glove surface, the approach is readily adaptable to a wide range of materials and environments. With minor optimization, the procedure could be refined to enhance viral recovery across different surface types, broadening its applications.

## 3 Conclusion

This work presents the development of a rapid and portable electrochemical sensor for norovirus detection, based on an electropolymerized MIP. The sensor demonstrated consistent detection across multiple norovirus VLPs genotypes, with LoDs in the femtogram range. The swab‐based sampling approach offers a practical route for norovirus testing by streamlining sample preparation. Incorporating gloves as a test surface extends the method's applications to both healthcare and food handling environments, supporting proactive measures to limit transmission. Importantly, the process from sample collection to electrochemical read‐out was completed in 15 min. The sensor exhibited specificity demonstrated by negligible binding to a nontarget imprinted control polymer, and high selectivity against other prevalent viruses, including HCoV‐299E, HSV‐2, RSV, and CMV. Furthermore, the electrodes maintained binding performance following three months of storage under ambient conditions, supporting the feasibility of storage and distribution without cold‐chain requirements. In this work, the integration of a smartphone with a compact potentiostat keeps the total device weight below 200 g, enhancing portability. Moreover, the system operates without the requirement of access to electrical outlets, enabling measurements to be performed in remote or resource‐limited settings, therefore improving overall accessibility. The use of low‐cost SPGEs significantly reduces the cost per test compared to traditional gold or platinum electrodes, whilst also eliminating the need for separate counter and reference electrodes. Combined with the inexpensive pyrrole monomer and minimal target protein required for MIP synthesis, the overall platform offers a cost‐effective alternative to conventional methods. Collectively, these results define a rapid and robust sensing platform for on‐site norovirus detection, enabling more responsive contamination control and mitigation of viral transmission.

## 4 Materials and Methods

### 4.1 Materials

Pyrrole (Cat. No. 131709), potassium ferricyanide (III) (Cat. No. 702587) and potassium hexacyanoferrate (II) trihydrate (Cat. No. P9387) were purchased from Sigma‐Aldrich (Gillingham, UK). Potassium chloride (KCl) (Cat. No.), PBS (Oxoid PBS tablets Cat. No. BR0014G for electrochemistry experiments, Sigma Life Science D8537‐500 mL for cell culturing), hydrochloric acid 33% (HCl) (Cat. No. 010990.AU), sodium hydroxide (NaOH) (Cat. No. A16037.36) and nitrile powder‐free protective gloves were purchased from Fisher Scientific (Loughborough, UK). Recombinant norovirus GI.1 (Cat. No. REC31722), GII.2(Cat. No. REC31983) and GII.17 (Cat. No. REC31989) VP1 VLPs (100 µg at >90% purity) were purchased from the Native Antigen Company (Kidlington, UK). Recombinant norovirus GII.4 VP1 VLPs (Cat. No. ab256447) with purity >95% were purchased from Abcam (Cambridge, UK). All experiments utilized deionized water (DI) with a resistivity of ≥18.2 MΩ cm. HSV‐2, CMV, HCoV‐299E, and RSV were provided by the University of Birmingham.

Electrochemical measurements were performed using a Sensit Smart potentiostat (PalmSens, Houten, The Netherlands) controlled by PSTrace 5.11 / PStouch software on a Huawei P30 Android smartphone or laptop.

Electrochemical measurements were performed using a Sensit Smart potentiostat (PalmSens, Houten, The Netherlands) controlled by PSTrace 5.11 / PStouch software on a Huawei P30 Android smartphone or laptop.

### 4.2 P Domain Synthesis

The norovirus P domain from norovirus GII.4 2006b was cloned based upon previous work [49]. *E. coli* BL21 (DE3) was used for pGEX‐4T‐1/GII.4_P domain transformation and cultured in 1 L 2 × YT supplemented with ampicillin (150 µg/mL) at 37°C to an OD600 of 0.6. The cells were induced by 0.25 mM isopropyl β‐d‐1‐thiogalactopyranoside and incubated with shaking at 23°C overnight. They were harvested by centrifugation at 3184 rcf for 20 min using Eppendorf 5810R (Eppendorf, MA, USA), resuspended and lysed in 1 × PBS containing 0.1 mM phenylmethylsulfonyl fluoride and 1 mM ethylenediaminetetraacetic acid. The lysate was centrifuged at 13,000 rpm for 15 min at 4°C using an Avanti JXN‐26 (Beckman Coulter, Inc., CA, USA) with a JA‐25.50 rotor, and the supernatant was used for subsequent purification steps. The P domain was first purified using Glutathione Sepharose 4B (Cytiva, MA, USA) resin in a gravity‐flow column. For additional polishing, the protein was subjected to gel filtration chromatography (GFC) on a Superdex 200 column (GE Healthcare, IL, USA) using a fast protein liquid chromatography system (Cytiva, MA, USA), with 1 × PBS as the mobile phase at a flow rate of 0.5 mL/min.

### 4.3 Production of Screen‐Printed Graphite Electrodes

SPGEs were fabricated using a stencil‐based design to produce a working electrode with a 3.1 mm diameter [50]. Fabrication began with the deposition of graphite ink onto a flexible polyester substrate using a DEK 248 screen‐printing system. The printed graphite layer, forming the working electrode, was then thermally cured in a fan‐assisted oven at 60°C for 30 min. Subsequently, a silver/silver chloride (Ag/AgCl) paste was screen‐printed to define the reference electrode and cured under the same conditions. Finally, a commercial dielectric insulating layer (Product code: D2070423D5, Gwent Electronic Materials, Pontypool, United Kingdom) was applied over the conductive tracks to prevent cross‐talk between electrodes, followed by an additional 30 min curing step to complete the assembly. Due to the use of a mechanized automated screen printer with set print gaps of 1.65 mm, constant print speeds and identical screens, the SPEs are manufactured with high reproducibility both intra (3.5%, n = 28)—and inter‐batch (4.9%, n = 5). These values show good agreement with reported work on SPEs using automated screen printing, which identifies ranges of deviation between 2%–10% [51].

### 4.4 Electropolymerization

The SPGEs were first cleaned by ultrasonication (10 min) in DI water and left to dry at room temperature. The monomer solution was prepared containing 0.1 M pyrrole in PBS containing 60 µg/mL of the P domain. The electrodeposition of the polypyrrole layer was performed by adding 60 µL of the monomer solution to the surface of the electrode and running chronoamperometry at a potential of +0.8 V (vs. Ag/AgCl) for 180 s. Using the same parameters, a control polymer was produced using 60 µg/mL of bovine serum albumin (BSA) instead of the P domain. The modified SPGE was then kept in a solution of 0.1 M HCl (30 min) for template extraction and then rinsed with DI water. This was then placed into a 1% solution of BSA for 15 min.

### 4.5 Characterization

Surface chemical analysis was carried out by XPS spectroscopy using an AXIS Supra instrument (Kratos, UK). Spectra were acquired with a monochromatic Al Kα X‐ray source (1486.6 eV) operated at 225 W and analyzed using a hemispherical sector analyzer in fixed transmission mode. Spectra were collected at a pass energy of 160 eV, while high‐resolution scans were conducted at 20 eV. Under the high‐resolution acquisition conditions, the Ag 3d_5_/_2_ peak gave a full width at half maximum of 0.613 eV. Binding energies were charge‐corrected to the sp^2^ C1s peak at 284.5 eV. Although this referencing method has recognized limitations [52], it was selected because no more suitable internal reference was available and the interpretation did not rely on absolute binding‐energy values.

SEM was used to examine sample morphology. Images were acquired on a Crossbeam 350 FIB‐SEM instrument (Carl Zeiss Ltd, Cambridge, UK) fitted with a field‐emission electron source. Secondary electron images were recorded using a Secondary Electron Secondary Ion detector. Prior to analysis, specimens were fixed onto 12 mm aluminum SEM pin stubs with 12 mm adhesive carbon tabs, both from Agar Scientific (Essex, UK). The mounted samples were then coated with a 5 nm Au/Pd conductive layer using a Leica EM ACE200 sputter coater before imaging.

### 4.6 Electrochemical Measurements

All electrochemical measurements were recorded using a redox solution of 5 mM [Fe (CN)_6_] ^−3/−4^ with 0.1 M KCl. CV measurements were performed using a potential range of +0.7 to ‐0.4 V (vs. Ag/AgCl) at a scan rate of 50 mV/s. The SWV parameters were set as follows: an amplitude of 0.25 V, a step potential of 0.05 V, and a frequency of 1 Hz, over a potential range of ‐0.2 V to 0.6 V (vs. Ag/AgCl). For CV measurements, a broad concentration range of 1 to 10^5^ fg/mL was explored, corresponding to 5.7 × 10^1^ to 5.7 × 10^6^ particles/mL. This range was applied to the P domain and four norovirus VLPs. The selectivity viruses were performed using concentrations between 10^3^ and 10^5^ PFU/mL. For SWV measurements, the concentration range was narrowed to 50 to 1000 fg/mL, corresponding to 2.9 × 10^3^ to 5.7 × 10^4^ particles/mL. Sample measurements were carried out by incubating 10 µL of the sample solution on the SPE for 10 min, followed by rinsing with DI water and gentle drying under nitrogen. Subsequently, 60 µL of the redox solution was added to the SPGE, and the measurement was performed. The LoD for each of the targets was calculated by utilizing the three‐sigma method, shown in equation S1.

### 4.7 Nontarget Virus Production

HSV‐2, CMV, (HCoV‐299E—samples originally isolated, verified, and donated by the School of Biological Sciences, University of Manchester) and RSV were produced as nontarget viruses for selectivity studies.

#### 4.7.1 Cell Culture

All cell lines were maintained in T75 tissue culture flasks and grown in Dulbecco's Modified Eagle Medium (DMEM; Vero cells (CCL81), F12K, 2 mM glutamine; A549 cells (CCL‐185), Eagle's Minimum Essential Medium (EMEM) with Earle's Balanced Salt Solution (EBSS), 2 mM Glutamine, 1% Nonessential Amino Acids (NEAA); HeLa cells) supplemented with 10% fetal bovine serum (heat inactivated, Thermo Fisher Scientific Gibco FBS qualified heat inactivated Cat no: 10500064) and 1% penicillin‐streptomycin (Sigma Life Science, Cat no:P4333‐100ML). Cultures were incubated at 37°C in a humidified atmosphere containing 5% CO_2_. Cells were passaged as required and maintained until they reached approximately 80%–90% confluency before being used for subsequent experiments.

#### 4.7.2 Virus Expansion of CMV, RSV, and HCoV‐299E

For general viral propagation, confluent monolayers of host cells were infected with virus inoculum and incubated until a cytopathic effect (CPE) of approximately 90% was observed. At this stage, both the infected cells and the culture supernatant were collected using a sterile cell scraper and transferred into 15–50 mL conical tubes. The cell suspension underwent three freeze–thaw cycles to facilitate viral release through cell lysis. The resulting lysate was centrifuged at 5,000 rpm for 10 min at 4°C to remove cell debris. The clarified supernatant containing the viral particles was then aliquoted into 1 mL cryovials, snap‐frozen in liquid nitrogen, and stored at −80°C until required for downstream applications.

#### 4.7.3 Production of Herpes Simplex Virus 2 (HSV2)

HSV‐2 was propagated using Vero cell lines following a standardized procedure adapted from conventional virological methods. Briefly, confluent T75 flasks of Vero cells were prepared under standard culture conditions. Once full confluency was achieved, the culture medium was removed and replaced with 5 mL of serum‐free medium (DMEM). The virus inoculum was then added to the flask to initiate infection and incubated for 2 h at 37°C to allow viral adsorption. After incubation, the inoculum was removed, and 12 mL of fresh serum‐free medium was added. The infected cultures were maintained until approximately 95% cytopathic effect (CPE) was observed. At this stage, both the cells and the culture medium were collected using a sterile cell scraper and transferred into a 15 mL conical tube. The sample was centrifuged at 5,000 rpm for 10 min at 4°C to separate the supernatant from the cell pellet. The supernatant was transferred to a clean tube and kept on ice, while the pellet was resuspended in 500 µL of the collected supernatant. The resuspended pellet underwent three freeze–thaw cycles (freezing at −80°C for 10 min and thawing at room temperature) to ensure complete viral release. The lysed sample was centrifuged again at 5,000 rpm for 10 min at 4°C, and the resulting supernatant was pooled with the previously collected one. The combined viral suspension was mixed gently by inversion, aliquoted into 1.5 mL microcentrifuge tubes (800 µL per aliquot, approximately 48 in total), and stored at −80°C for long‐term preservation.

#### 4.7.4 Virus Titration

Viral titers were quantified using a standard plaque assay to determine the concentration of infectious viral particles, expressed as PFU/mL. Host cells were seeded into 24‐well tissue culture plates at a density of 8.5 × 10^4^ cells per well and incubated overnight at 37°C with 5% CO_2_ to allow for monolayer formation.

Serial 10‐fold dilutions of the viral stock were prepared, and appropriate dilutions (producing between 50 and 100 plaques) were used to infect the cell monolayers. Following incubation and plaque development, plaques were counted manually, and viral titers were calculated using equation 1 below:

(1) $$\begin{array}{cc} & \text{PFU}/\text{mL}\\ & \;=(\text{Number}\;\text{of}\;\text{plaques}\times \text{Dilution}\;\text{factor})/\\ & \;\text{Volume}\;\text{of}\;\text{inoculum}(\text{mL}) \end{array}$$

The viruses used in this study included CMV, HSV‐2, RSV, and HCoV‐229E, each with stock concentrations exceeding 10^7^–10^8^ PFU/mL. The cell lines utilized for virus propagation and titration included Vero, Hela, and A549 cells.

### 4.8 Swab Samples

The swab sampling protocol was adapted from Rönnqvist et al. [53]. A 25 cm^2^ section of a nitrile glove was used as the sampling area and was evenly inoculated with 100 µL of the sample dilution. These were left overnight to dry in the fume hood. Prior to swabbing, 1 mL of PBS was added to the tube to moisten the swab. The test area was then swabbed for 1 min, after which the swab was rotated, and this was repeated on the opposite side. The swab was then returned to the tube and placed onto the orbital shaker for 5 min before being used for analysis.

### 4.9 Statistical Analysis

All electrochemical measurements were performed using three independent replicates (n = 3). Data preprocessing included baseline correction of SWV signals prior to determining peak current values. Change in current (ΔI) was determined by subtracting the baseline signal from the corresponding analyte response signal. The baseline signal was defined as the MIP modified SPGE before analyte addition. Dose‐response curves were plotted as the ΔI against the analyte concentration. Data are presented as mean ± standard deviation (SD). For dose‐response curves, circular symbols or bars represent the mean of three independent measurements, while error bars indicate SD. Paired T‐tests were used to compare the sensor under numerous conditions at matched concentrations. Statistical significance was defined as p < 0.05. Assumptions of normality and equal variance were considered acceptable due to the consistency observed across replicate measurements. All statistical analyses and graph preparation were performed using GraphPad Prism, with illustrations prepared using Blender.

## Author Contributions

AD: Conceptualization, Methodology, Investigation, Formal Analysis, Data Curation, Visualization, Writing – Original Draft. PS: Methodology, Investigation, Formal Analysis, Writing – Review & Editing. MK: Methodology, Investigation, Resources, Writing – Review & Editing. SS: Methodology, Investigation, Resources, Writing – Review & Editing. CT: Methodology, Investigation, Resources, Writing – Original Draft. RDC: Methodology, Resources, Writing – Original Draft. CEB: Resources, Supervision, Writing – Review & Editing. JMC: Methodology, Investigation, Supervision, Writing – Review & Editing. SS: Supervision, Writing – Review & Editing. MG: Supervision, Writing – Review & Editing. STJ: Resources, Supervision, Writing – Review & Editing. CFB: Resources, Supervision, Writing – Review & Editing. MDM: Conceptualization, Resources, Supervision, Funding Acquisition, Project Administration, Writing – Review & Editing. MP: Conceptualization, Supervision, Project Administration, Funding Acquisition, Writing – Review & Editing.

## Conflicts of Interest

The authors declare no conflicts of interest.

## Supporting information




**Supporting File**: adhm71467‐sup‐0001‐SuppMat.docx.

## Data Availability

The data that support the findings of this study are available in the Supporting Information of this article and from the corresponding author upon request.
