# Supplementary material for: Portable Molecularly Imprinted Polymer Sensor for Rapid Swab‐Based Detection of Norovirus
Source: Adv Healthc Mater. 2026 Jul 21;15(31):e71467. doi: 10.1002/adhm.71467 (PMC13495804; doi:10.1002/adhm.71467)
Supplement: Supplementary file 1 [file ADHM-15-0-s001.docx]

# Supplementary information

Table S1: Atomic concentration of polypyrrole layer calculated from XPS analysis

| Group | Atomic Concentration % |
| --- | --- |
| C=C | 11.3 |
| C-C, C-H | 53.83 |
| C-O, C-N | 20.31 |
| C=O, C=N | 9.25 |
| N-C=O, O-C=O | 3.79 |
| Pi-Pi | 1.52 |

Figure S1: XPS spectra of the polypyrrole-modified electrode showing a) survey scan indicating the presence of C, N and O elements and high-resolution spectra of b) C 1s, c) O 1s, and d) N 1s regions.

LoD $=\frac{3\sigma}{S}$	Equation S1

where σ is the standard deviation of the baseline of a blank concentration of analyte, and S is the slope of the dose-response curve.

Figure S2: Dose-response of the sensor to increasing P domain concentrations after electrode storage under ambient conditions for various time intervals (same day, 1 week, 1 month and 3 months). The response is presented as mean ± standard deviation (µA per log_10_[concentration]), n=3. Circular symbols represent the mean of three independent replicates, with error bars showing the standard deviation of the measurements.

Figure S3: a. Example square wave voltammogram upon additions of increasing concentrations of P domain. b. Dose-response curves showing the ΔI upon the addition of P domain concentrations using SWV. The response is presented as mean ± standard deviation (µA per log_10_[concentration]), n=3. Circular symbols represent the mean of three independent replicates, with error bars showing the standard deviation of the measurements. Colored lines indicate the linear fit to the data, and dotted lines represent the 95% confidence interval.

Table S2: Calculated LoD values for each of the targets using CV, SWV and swab samples

| Analyte | LoD (fg/mL) | | |
| --- | --- | --- | --- |
|  | CV | SWV | Swab |
| P Domain | 49 | 227 | N/A |
| GII.4 VLP | 106 | 153 | 154 |
| GII.2 VLP | 182 | 163 | 279 |
| GII.17 VLP | 210 | 174 | 208 |
| GI.1 VLP | 151 | 158 | 208 |

Table S3: Characteristics of norovirus, human coronavirus 299E (HCoV – 299E), herpes simplex virus serotype two (HSV-2), respiratory syncytial virus (RSV), and cytomegalovirus (CMV).

| **Virus** | **Genome type** | **Family/Genus** | **Size (nm)** | **Capsid Symmetry** | **Enveloped** | **Associated illness** | **Ref.** |
| --- | --- | --- | --- | --- | --- | --- | --- |
| Norovirus | Positive-sense RNA | Caliciviridae/  Norovirus | ~40 | Icosahedral | No | Gastroenteritis | ^49^ |
| HSV-2 | DNA | Herpesviridae/  Simplexvirus | ~110 | Icosahedral | Yes | Genital herpes | ^50^ |
| CMV | DNA | Herpesviridae/ | ~150 -200 | Icosahedral | Yes | Mononucleosis-like illness | ^51^ |
| HCoV- 299E | Positive-sense RNA | Coronaviridae/ Alphacoronavirus | ~80 -120 | Helical | Yes | Upper respiratory tract infection | ^42^ |
| RSV | Negative-sense RNA | Pneumoviridae/  Orthopneumovirus | ~120 - 300 | Helical | Yes | Upper respiratory tract infection | ^52,53^ |

Table S4: Paired t-test results comparing the sensor response of the norovirus genotypes to each of the selectivity viruses. P values are expressed as P<0.05 (*) , P<0.01(**)

| Virus response comparison | T-test P value, Significance | | | |
| --- | --- | --- | --- | --- |
|  | GII.4 | GII.2 | GII.17 | GI.1 |
| HSV-2 | 0.0025,** | 0.0025,** | 0.0019,** | 0.0021,** |
| CMV | 0.0037,** | 0.0044,** | 0.0031,** | 0.0039,** |
| HCoV-299E | 0.0042,** | 0.0050,** | 0.0037,** | 0.0045,** |
| RSV | 0.0038,** | 0.0042,** | 0.0032,** | 0.0036,** |
